# Repair of a defect in the cervical trachea with thyroid-pericardium flap: A case report

**DOI:** 10.1097/MD.0000000000017871

**Published:** 2019-11-15

**Authors:** Hui Xie, Yuqian Zhang, Fenglei Yu, Xiang Wang

**Affiliations:** Department of Thoracic Surgery, Second Xiangya Hospital of Central South University, Changsha, China.

**Keywords:** thyroid-pericardium patch, tracheal diseases, tracheoplasty

## Abstract

**Rationale:**

Primary end-to-end anastomosis is common in adult trachea resection. Nevertheless, considering that the utilization of grafts is still essential for restoring defect in long-segment tracheal resection surgery, long-segment tracheal resection and reconstruction still remain challenging. Herein we present a novel case in which we resected a large tracheal mass and reconstructed the long-segment defect through using a thyroid-pericardium flap, which has not been reported yet.

**Patient concerns:**

A 35-year old male patient was admitted due to ‘Repeated dry cough for 2 years, shortness of breath after activities for 1 month’. Patient had no other obvious symptoms.

**Diagnoses:**

CT revealed that a large neoplasm was located in the cervical trachea and the pedicle was in the tracheal membrane, with total length of approximately 6 cm. Positron emission tomography computed tomography demonstrated an abnormally elevated levels of glucose metabolism in the upper part of the posterior tracheal wall. Therefore, this lesion was primarily considered as a malignancy.

**Interventions:**

The patient was performed by a primary resection of long-segment tracheal mass followed by thyroid-pericardium composite tissue flap for reconstruction.

**Outcome:**

The operation was successful, without hydrops or pneumatosis in the mediastinum. One week postoperatively, CT showed that there was no pneumomediastinum and mediastinal abscess. Three weeks postoperatively, fiber bronchoscope showed the flap with normal color and the unobstructed tracheal cavity. The patient healed without complication.

**Lessons:**

The thyroid-pericardium flap is a convenient, secure, and effective material for long-segment trachea mass resection and reconstruction.

## Abstract 2

Supplemental Digital Content is available in the text

## 1 Introduction

Tracheal resection and primary end-to-end anastomosis is one of the most common surgical interventions for tracheal neoplasms. Nevertheless, some reports described that the maximum length of tracheal resection that can be repaired by end-to-end anastomosis is merely 6 cm,^[1]^ which makes the long-segment tracheal resection and reconstruction challenging for surgeons. Therefore, researchers have been showing a keen interest in exploiting the safe, effective and accessible grafts that are sufficient to restore the defect caused by long-segment tracheal resection. Etienne et al proposed that 5 types of grafts were in common use, including synthetic prosthesis, allografts, tracheal transplantation, tissue engineering, and autologous tissue composite.^[2]^ However, the ideal tracheal substitutes do not currently exist, as all of the substitutes are not well vascularized, rigid and autologous enough.^[3]^ We present the first successful utilization of a thyroid-pericardium flap to reconstruct the tracheal defect caused by long-segment resection in a patient with a large tracheal mass. And the patient has provided informed consent for publication of this case.

## 2 Case presentation

**Figure 1:**
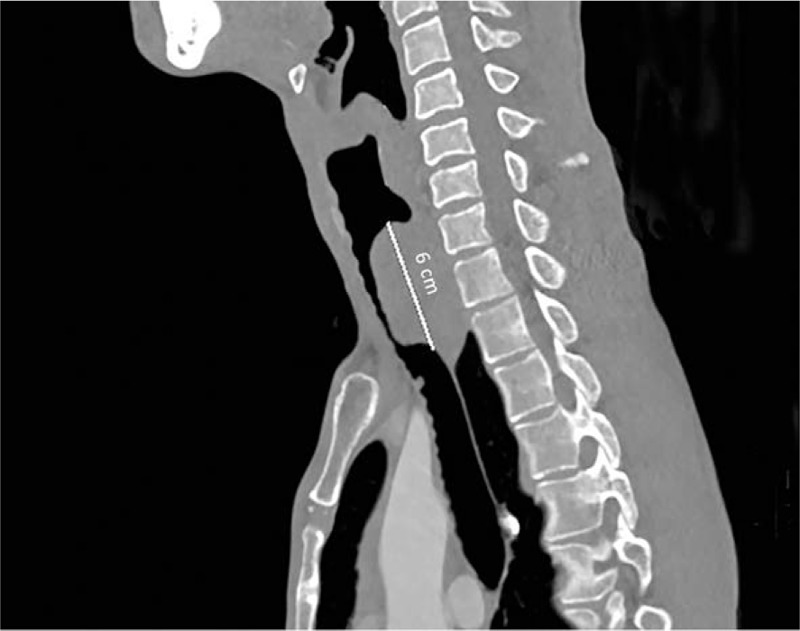
CT scan: occupying lesion in the cervical trachea, the pedicle was in the tracheal membrane, total length was about 6 cm.

**Figure 2:**
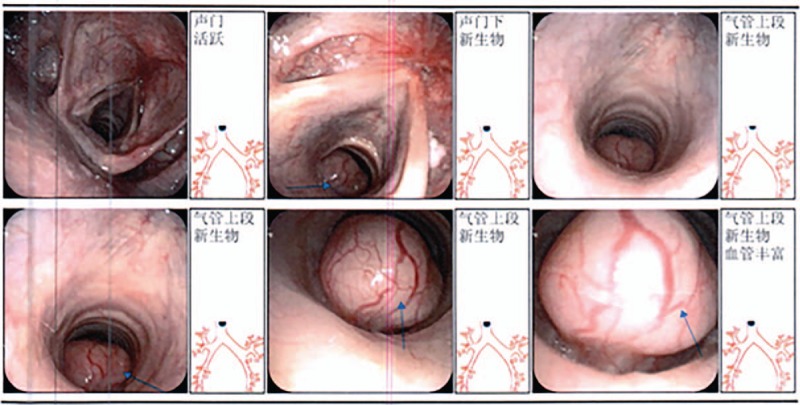
Fiber bronchoscope showed: a spherical neoplasm can be seen in upper part of trachea which was 1.5 cm under the glottis, it was eulogistic and the surface was smooth, the neoplasm had a pedicle and cannot be moved. The mass protruded to the lumen, resulting in annular narrow of the upper trachea, distal end cannot be peeked.

A 35-year old male patient was admitted into hospital owing to a newly discovered tracheal masses, the chronic and repeated dry cough and the shortness of breath after activities. CT revealed a space-occupying lesion in the cervical trachea. The pedicle was in the tracheal membrane and displayed a total length of about 6 cm (Fig. 1). Fiber bronchoscope showed that a spherical neoplasm could be seen in the upper part of the trachea and was 1.5 cm below the glottis. Additionally, this neoplasm was characterized by hypervascularity, smooth surface and failure to be moved. The mass protruded to the lumen, resulting in annular narrow of the upper trachea. Its distal end could not be observed (Fig. 2). Positron emission tomography computed tomography revealed a remarkably increased levels of glucose metabolism in the upper part of the posterior tracheal wall. Esophagoscope showed that esophagus was not invaded by the mass. Collectively, this lesion was considered as a malignancy. Patient had no other severe diseases and did not undergo any surgery in the past.

The patient was scheduled for a primary resection of long-segment tracheal mass and reconstruction by using autologous thyroid-pericardium composite tissue flap. Intraoperatively, the patient was in the supine position. A cervically endotracheal mass with about 6 cm of length was seen following conducting a neck and chest midline incision. The mass extended ranging from the place of 1.5 cm below the glottis to the thoracic inlet level of trachea. Furthermore, the mass was obviously invasive outward and bulged nearly 2 cm outward the tracheal membrane, indicating that it appeared to be closely anatomically associated with the right recurrent laryngeal nerve. It also encroached the part of fibrous membrane and muscular layer of the right wall of cervical segment of esophagus. There was an enlarged cervical lymph node, with moderate hardness and no tendency of invasion.

**Figure 3:**
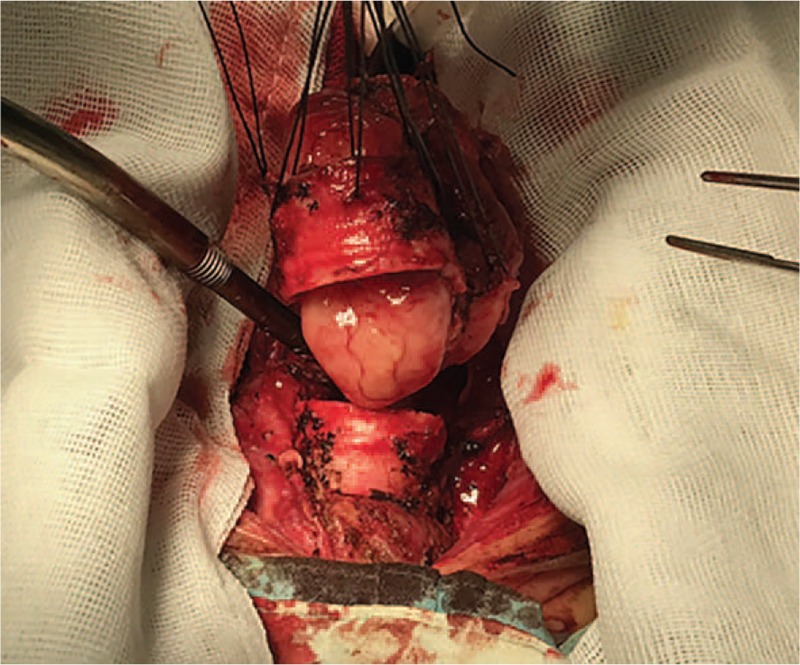
The middle of trachea contained tumor was resected which involved in about 4 tracheal cartilages.

**Figure 4:**
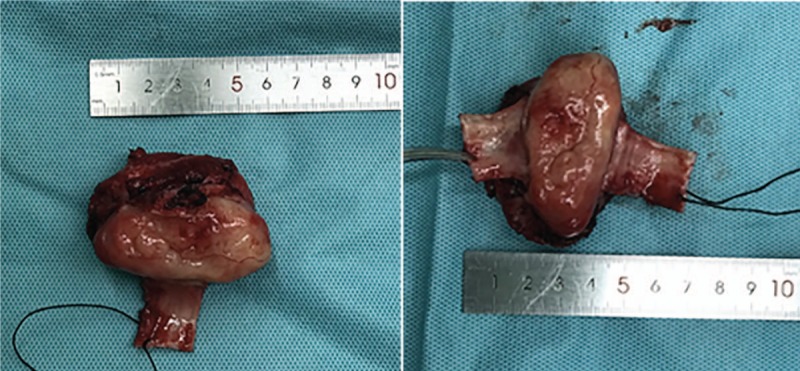
Measurement of the tumor resected.

**Figure 5:**
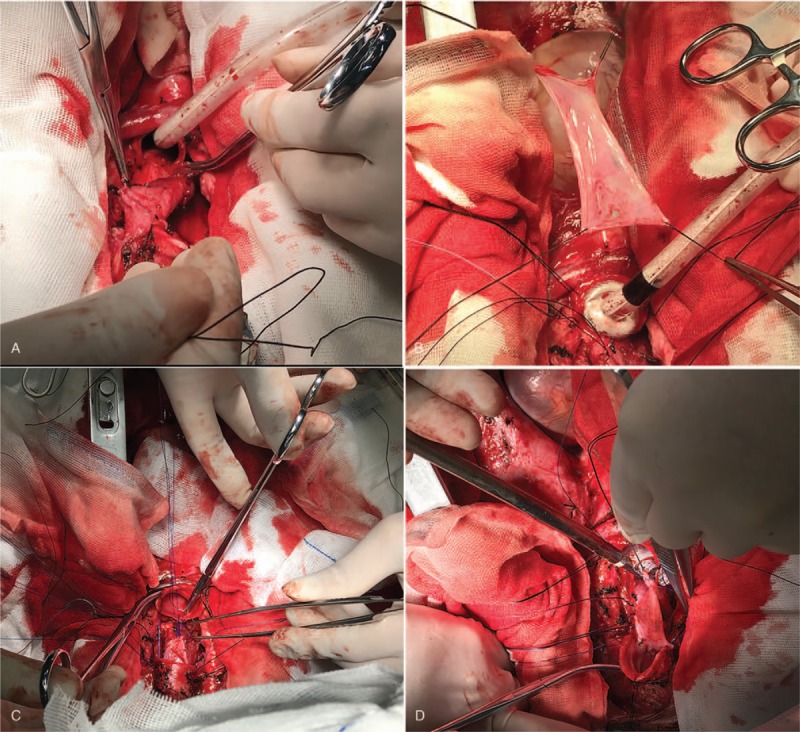
(A) Left thyroid was relocated to the anterior of cervical vertebra, (B) part of pericardium was resected, (C, D) pericardium and thyroid gland were sutured together to make into a composite flap.

**Figure 6:**
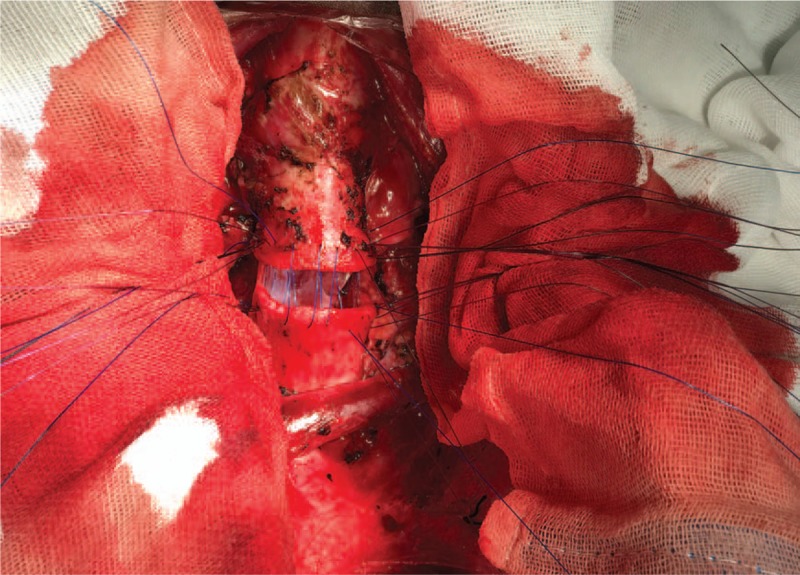
Suture tracheal membrane by 2-0 absorbable line, tracheal cartilages of anterior and lateral walls was sutured by 3-0 monocryl.

Initially, the thymus was resected to expose the trachea above the innominate artery that was near the thoracic inlet level. The bilateral recurrent laryngeal nerves were exposed and the right recurrent laryngeal nerve was in a closely anatomical relationship with the tumor. Thus, it was pivotal to protect right recurrent laryngeal nerve from mechanical injury. Then, the tumor in conjunction with involved trachea (including about 4 tracheal cartilages of the anterior and lateral wall) were resected (Fig. 3), and the posterior wall was also resected about 6.5 cm (Fig. 4). Two stumps and the inferior margin of tracheal membrane were taken for an urgent pathological examination and there were no remnant tumor cells. The anterior wall of trachea could be closed with low tension. The defective region of trachea posterior wall was approximately 2 × 3 cm^2^. The right isthmus and isthmus of the thyroid were isolated to be protected. Left thyroid arteries and veins were reserved and the right side of the thyroid was relocated to the anterior of the cervical vertebra. The part of the pericardium was resected and its smooth surface face was toward the inner side of the trachea. Ultimately, the pericardium and thyroid gland were sutured together and were made into a composite flap (Fig. 5). The defect of the membrane was repaired with flap by running suture of 3-0 monocryl, and the anterior and lateral walls were reconstructed by 3-0 monocryl (Fig. 6). The pericardial defect was repaired by bovine pericardium patch.

**Figure 7:**
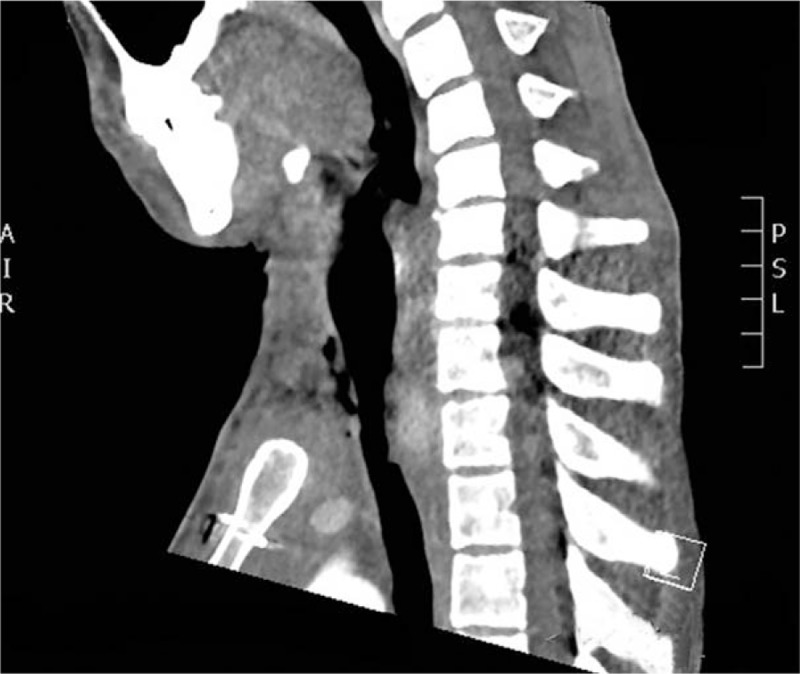
CT scan 8 days after operation.

**Figure 8:**
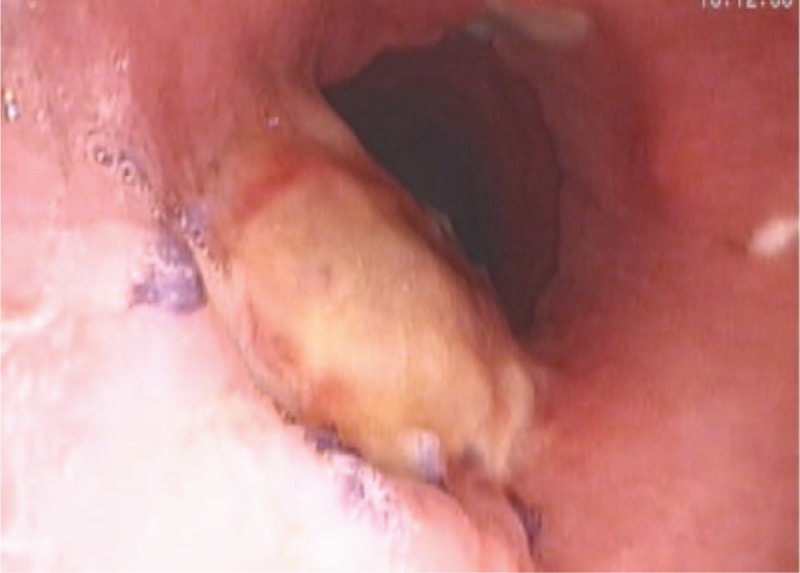
Fiber bronchoscope 15 days after operation.

Eight days postoperatively, CT scan showed there were no pneumomediastinum and mediastinal abscess (Fig. 7). The pathological examination demonstrated that this tumor was derived from epithelial cells and tumor cells were characterized by adenoid or cribriform structures. According to immunohistochemistry, it was considered as highly differentiated adenoid cystic carcinoma with the capacity to invade bronchial wall. The results of immunohistochemistry were the followings: Ki-67(30%), CK5/6(+), P40(+), CK7(+), CAM5.2(+), Syn(–), CgA(–), s100(+), TTF-1(–), P53(20%+), P63(+), CD117(+). Two weeks postoperatively, the patient recovered better and was discharged from hospital. Three weeks postoperatively, fiber bronchoscope showed the flap was in normal color and the tracheal cavity was unobstructed (Fig. 8). More media presentation can be seen in supplementary multimedia.

## 3 Discussion

In this case, the patient was endowed with a large mass in the trachea, tracheal end-to-end anastomosis was not a viable choice. Some reports described the maximum length of tracheal resection that can be resected and repaired by end-to-end anastomosis was 6 cm. The patient's tracheal defect in anterior wall could be closed with low tension, however, posterior wall defect of trachea was 2 × 3 cm^2^, which was difficult to sew up, so the flap came into our consideration.

Autologous tissue composites, such as muscle and pedicled myocutaneous flaps, have been used in many tracheal surgeries because of their strength and versatility,^[4,5]^ this technique needs preconcerted plan and changing the patient's position during surgery, so this preclude its use in emergency situations. Autologous pericardial patches as tracheal substitutes have the advantages of relatively high availability in the surgical field, easy accessibility, desirable pliability and tailoring to each case individually. Most importantly, they can give an airtight seal of the reconstructed airway, and confer robust resistance against infection.^[6]^ Fanous et al analyzed the postoperative effects of 26 patients with long-segment tracheal stenosis who underwent anterior pericardial tracheoplasty and further concluded that this kind of technology was sufficient to make contributions to the favorable prognosis of patients in the long term.^[7]^ Moreover, a few reports have described that the utility of a thymus pedicle for tracheal reconstruction was conducive to the success of the operation and the recovery of the patient a good result.^[8]^ In our case, patient's CT revealed space-occupying lesion in the cervical trachea, which was anatomically adjacent to the thyroid gland. Therefore, to reinforce the flap, we finally decided to use thyroid-pericardium flap.

Auchincloss et al reported that the complications occurred in about 20% of 589 patients after tracheal resection and reconstruction, such as granulation tissue formation, restenosis of the trachea, anastomotic separation, anastomotic fistula, wound infection, laryngeal edema, and glottic dysfunction and so on, which severely threatened the life of patients.^[9]^ The use of soft tissue such as single pericardium to replace large defects in the trachea can be problematic because of the lack of structural support in soft tissues, which can easily result in anastomotic fistula and separation. In our case, there were tremendous advantage through using thyroid-pericardium composites. Initially, patient did not suffer from the anastomotic fistula. the rigidity of the trachea reconstructed by thyroid-pericardium patch was satisfactory because we fixed thyroid gland onto cervical vertebra and further reinforced the flap. Furthermore, thyroid was readily available if the tracheal mass was near the cervical part of the trachea, which was extremely convenient Additionally, the patient did not suffer from any serious complications, suggesting a favorable safety profile of this technique- Therefore we think the thyroid-pericardium composite flap has both advantages of pericardium flap and thyroid gland flap.

In conclusion, we have shown that the thyroid-pericardium composite tissue flap can be easily and quickly used to repair and manage a defect in the cervical trachea, especially in long-segment trachea mass resection which grew eccentrically. This technique can be adopted for similar conditions, including some emergency situations.

## Author contributions

**Project administration:** Xiang Wang.

**Supervision:** Fenglei Yu, Xiang Wang.

**Validation:** Xiang Wang.

**Writing – original draft:** Hui Xie.

**Writing – review & editing:** Hui Xie, Yuqian Zhang.

Xiang Wang orcid: 0000-0002-5211-7206.

## Supplementary Material

Supplemental Digital Content
